# Prenatal Ultrasound Progression and Postnatal Retrospective Analysis of a Case of Small Intestinal Atresia with Volvulus

**DOI:** 10.3390/diagnostics16162525

**Published:** 2026-08-11

**Authors:** Lilu Nong, Lei Wang, Haiqiong Luo, Yan Wang, Chunchun Chen, Jiansheng Xie, Kanglin Dai, Jun Wang, Xiaohua Xu

**Affiliations:** 1 Department of Ultrasound, The University of Hong Kong-Shenzhen Hospital, Shenzhen 518053, China; nonglilu@cuhk.edu.cn (L.N.); wangl1@hku-szh.org (L.W.); wangy@hku-szh.org (Y.W.); 2 Department of Ultrasound, Shenzhen Medical Centre, The Chinese University of Hong Kong, Shenzhen 518172, China; 3 Prenatal Diagnosis Centre, The University of Hong Kong-Shenzhen Hospital, Shenzhen 518053, China; chencc@hku-szh.org (C.C.); xiejs@hku-szh.org (J.X.); 4 Shenzhen Clinical Research Center for Rare Diseases, Shenzhen 518053, China; 5 Department of Pediatric Surgery, The University of Hong Kong-Shenzhen Hospital, Shenzhen 518053, China; daikl@hku-szh.org; 6 Department of Pathology, The University of Hong Kong-Shenzhen Hospital, Shenzhen 518053, China; wangj@hku-szh.org

**Keywords:** prenatal ultrasound, small bowel atresia, small bowel volvulus

## Abstract

**Background and Clinical Significance:** Fetal jejunoileal atresia is a congenital anomaly of the lower digestive tract. Subsequent small intestinal volvulus, which may occur secondary to intestinal malrotation or atresia, represents a common cause of neonatal intestinal obstruction. The synthesis of intestinal atresia and volvulus is a dynamic, progressive process with distinct imaging features at each stage. Serial fetal ultrasound can detect abnormalities, and imaging changes may evolve from the initial onset of atresia to the development of volvulus. **Case Presentation:** We report the clinical data of a neonate diagnosed postnatally with small intestinal atresia complicated by volvulus, analyzed from a prenatal ultrasound perspective. The data include prenatal ultrasound findings, magnetic resonance imaging (MRI) results, postnatal ultrasound and computed tomography (CT) findings, intraoperative observations, pathological results, and clinical outcomes, followed by a retrospective analysis and discussion. In this case of small intestinal atresia with volvulus, prenatal ultrasound at 31 weeks of gestation revealed an enlarged stomach, proximal intestinal dilation, and collapse of the distal intestine (based on retrospective image review). At 35 weeks of gestation, a cystic mass resembling a “coffee bean sign” was detected in the fetal abdominal cavity (identified on retrospective image review), accompanied by polyhydramnios. Cesarean section was performed at 37 weeks, and the neonate underwent surgery on the second postnatal day. Intraoperative findings confirmed small intestinal atresia (Type IIIa) complicated by volvulus. The neonate recovered well postoperatively, and follow-up assessments indicated normal. **Conclusions**: Based on the retrospective analysis of this case, we propose that serial prenatal ultrasound and recognition of progressive imaging changes from intestinal atresia to volvulus carry important clinical significance. Multidisciplinary prenatal consultation plays a crucial role in formulating feasible postpartum treatment plans, thereby enhancing clinicians’ ability to manage such conditions effectively.

## 1. Introduction

Fetal small intestinal atresia complicated by volvulus represents an acute abdominal emergency stemming from a congenital gastrointestinal malformation. This condition can lead to intestinal perforation, meconium peritonitis, ascites, and anemia, and may even become life-threatening. Prenatal ultrasound diagnosis of this entity is highly challenging. Previously reported cases have predominantly focused on isolated intestinal atresia or intestinal malrotation, whereas reports of small intestinal atresia with concurrent volvulus are rare, particularly during the fetal period. In this case, the clinical data of the neonate were retrospectively analyzed, including the progression of prenatal ultrasound findings, magnetic resonance imaging (MRI) features, postnatal ultrasound findings, and computed tomography (CT) findings. These imaging characteristics were compared with the surgical and pathological findings. Improved recognition of the radiological features of this disease, together with dynamic changes seen on ultrasound, may allow a preliminary prenatal diagnosis of fetal small intestinal atresia with volvulus. Multidisciplinary prenatal counseling and selection of the optimal delivery timing may accordingly improve neonatal outcomes.

## 2. Case Presentation

**Figure 1:**
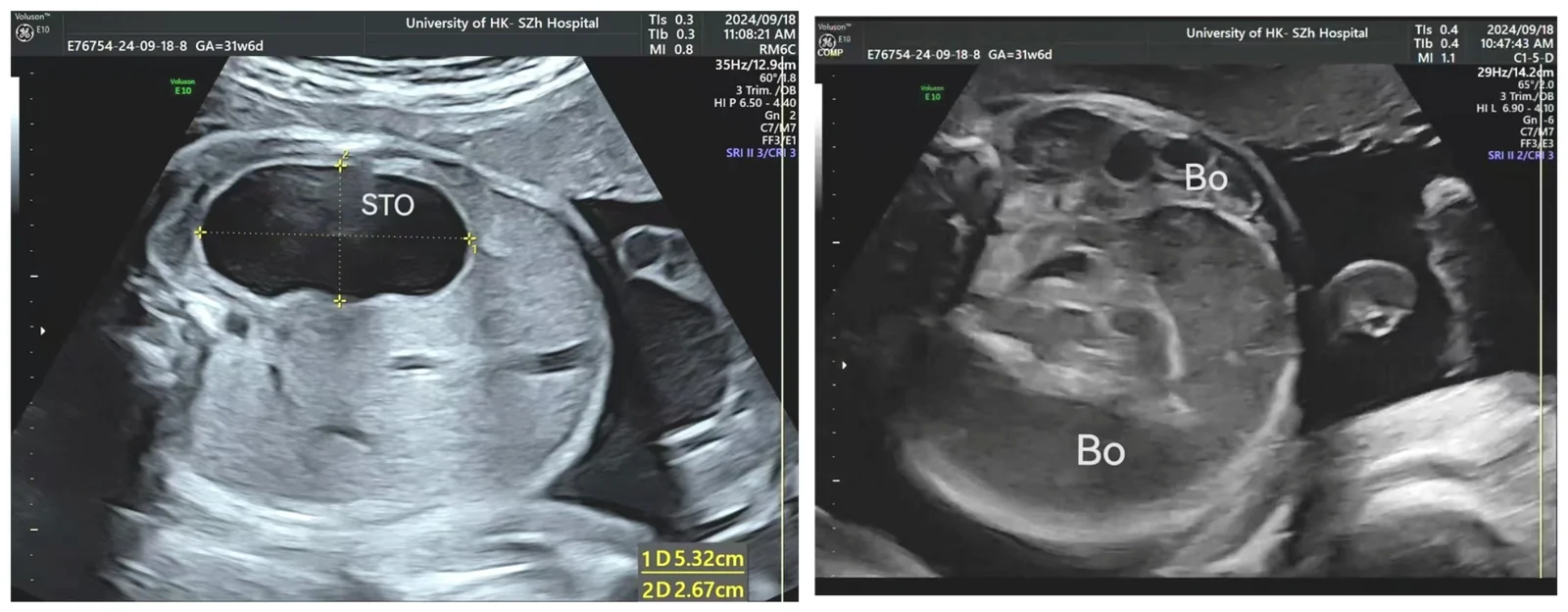
Fetal ultrasound (31 weeks): A large fetal stomach (**left image**) and a dilated fetal bowel (**right image**). STO: stomach; Bo: bowel. 1: anteroposterior diameter, 2: transverse diameter.

**Figure 2:**
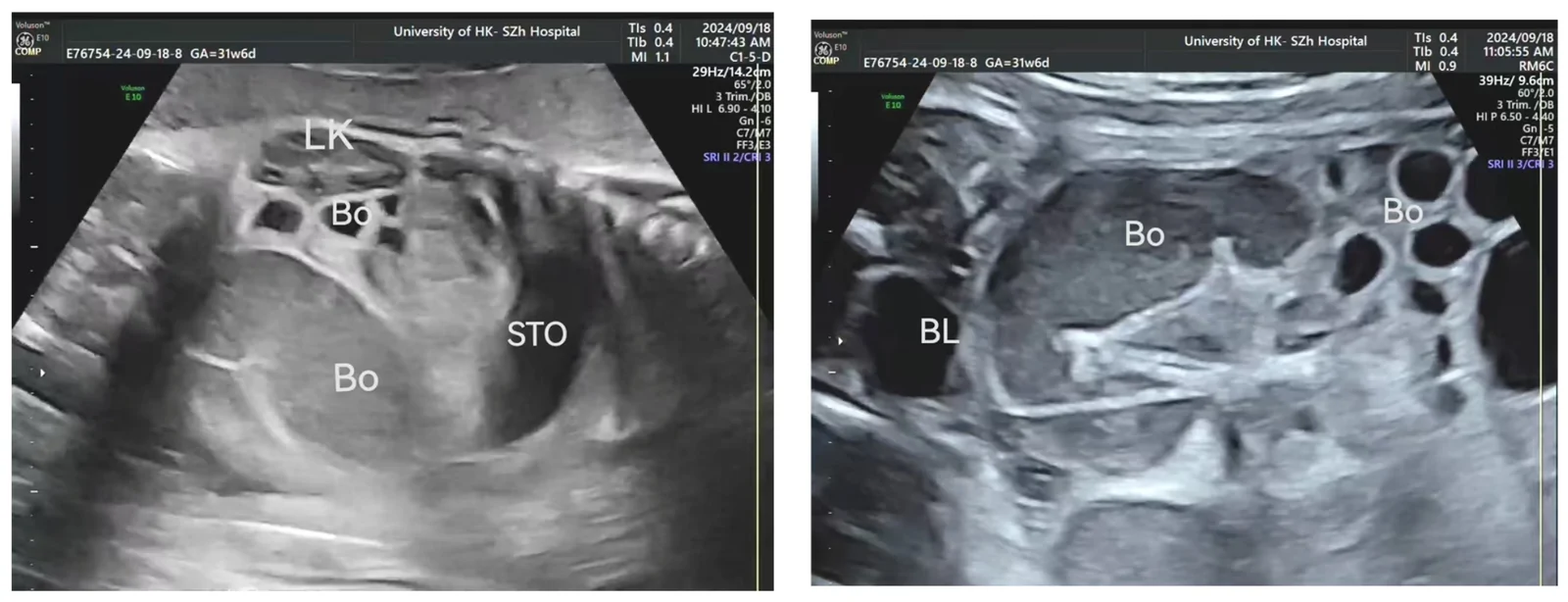
Fetal ultrasound (31 weeks): Longitudinal section (**left image**) and coronal section (**right image**) show dilated fetal bowel. STO: stomach; Bo: bowel; LK: left kidney; BL: bladder.

**Figure 3:**
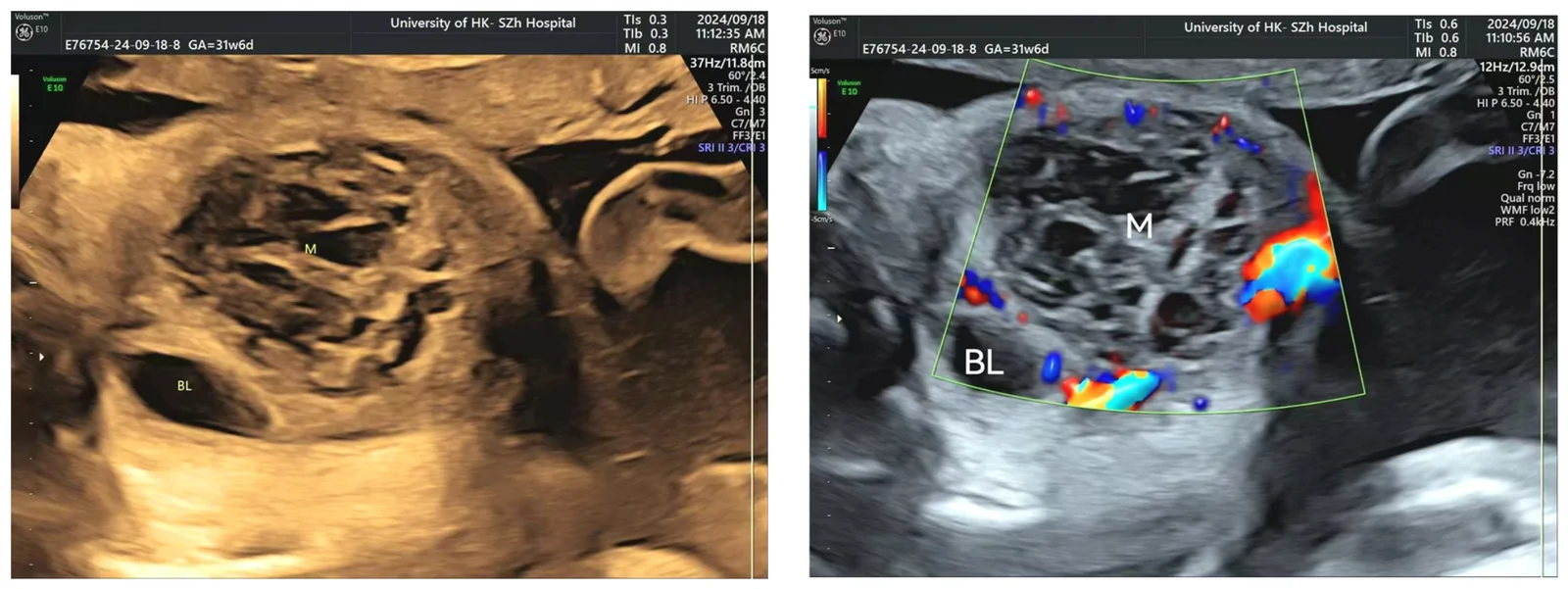
Fetal ultrasound (31 weeks): A mixed mass (postoperatively confirmed as a collapsed colon) is shown at the bottom of the pelvic cavity (**left image**). CDFI reveals point-line-like blood flow signals around the mass (**right image**). BL: bladder; M: mass.

**Figure 4:**
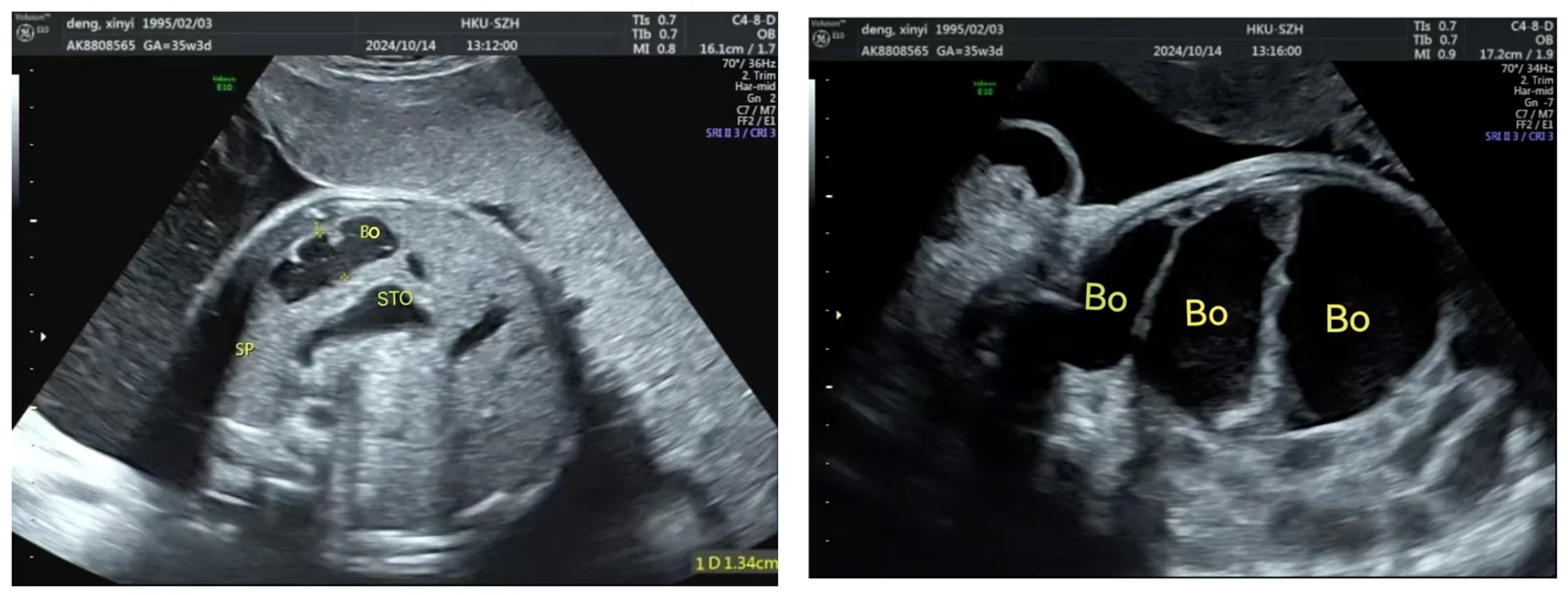
Fetal ultrasound (35 weeks): the fetal bowel appeared dilated in the (**left image**) and was markedly more dilated in the (**right image**); a cystic mass-like change resembling a “coffee bean sign” was observed in the fetal abdominal cavity (on retrospective image analysis). STO: stomach; Bo: bowel; SP: spleen. 1: lumen diameter.

The mother, a 29-year-old woman (gravida 2, para 1), had previously delivered a healthy male infant (birth weight: 3.5 kg) at 40 weeks of gestation via spontaneous vaginal delivery in 2022. The current pregnancy was conceived naturally. Nuchal translucency (NT) ultrasonography performed at 12 weeks of gestation showed an NT value of 1.6 mm, and the non-invasive prenatal testing (NIPT) result indicated low risk. Gestational diabetes mellitus was diagnosed in the second trimester, and blood glucose levels remained well-controlled throughout the pregnancy. At 22 weeks of gestation, fetal ultrasound examination showed that fetal biometry was consistent with gestational age, with no significant structural abnormalities and normal amniotic fluid volume. At 31 weeks of gestation, follow-up ultrasound revealed an enlarged stomach (53 × 27 mm) with dilatation of the small intestine and colon. The maximal luminal diameters were approximately 11 mm in the small intestine and 30 mm in the colon; however, postnatal surgical findings confirmed that the apparent colonic dilatation was not true colonic dilatation. The rectum was not significantly dilated, and the normal target sign of the anus was observed. On the transverse abdominal view, an irregular cystic mass measuring approximately 49 × 42 mm was noted in the left lower quadrant, later confirmed postoperatively to be a collapsed colon. The mass exhibited a honeycomb-like appearance with a relatively thick wall, and its echogenicity resembled that of the intestinal wall. Color Doppler flow imaging (CDFI) revealed linear blood flow signals along the wall of the mass. Amniotic fluid volume remained normal, with an amniotic fluid index (AFI) of 16.7 cm. Ultrasound findings indicated an intrauterine singleton live fetus in cephalic presentation, with size corresponding to approximately 31 weeks and 6 days of gestation. A large fetal stomach bubble and dilated bowel loops were observed, suggestive of lower gastrointestinal obstruction, though the underlying cause remains to be determined. An irregular cystic mass was noted in the lower fetal abdomen, with its origin and nature unclear; differential diagnoses include intestinal duplication cyst and other possibilities. Prenatal diagnostic consultation is recommended. Given the relatively early gestational age, fetal movements and monitoring remain normal. Continued observation is advised. Findings and Indications from Fetal Abdominal MRI at 34 Weeks of Gestation in Our Hospital: A large cystic mass measuring approximately 87 × 86 × 85 mm was identified in the mid-to-lower fetal abdomen, with a wall thickness of about 2 mm. Multiple strip-like and flocculent hypointense signals were observed within the mass. Its origin and nature remain uncertain. Dilated abdominal bowel loops were noted, with a maximum diameter of approximately 15 mm. The differential diagnosis includes an intestinal duplication cyst versus other cystic abdominal masses. Ultrasound Findings at 35 Weeks of Gestation: The stomach was not visualized with distension. Dilated intestinal loops were observed in the abdominal cavity, along with a cystic mass in the lower abdomen resembling a “coffee bean sign” (retrospective image analysis). Possible diagnoses include intestinal obstruction or Hirschsprung disease. An increased cardiothoracic ratio, mild tricuspid regurgitation, elevated diaphragm, bilateral testicular hydroceles, and polyhydramnios (amniotic fluid index, AFI: 32 cm) were also observed. The fetal ultrasound findings at 37 weeks of gestation were consistent with those described above. The antenatal ultrasound images are presented below (Figure 1, Figure 2, Figure 3 and Figure 4).

Amniocentesis at 31 weeks of gestation revealed no abnormalities on chromosomal microarray analysis (CMA), methylation analysis, or cytomegalovirus (CMV) DNA detection. Trio whole-exome sequencing (Trio-WES) was not performed. Clinical outcomes: At 35 weeks of gestation, following a discussion by the multidisciplinary team (MDT) comprising the Departments of Prenatal Diagnosis, Ultrasound, Neonatal Intensive Care Unit (NICU), and Pediatric Surgery, the following consensus was reached: fetal movements are currently normal, fetal heart monitoring is reassuring, and fetal growth parameters are consistent with gestational age. Premature delivery would increase the risk of preterm birth complications. If the fetal condition remains stable, an elective cesarean section is planned at 37 weeks of gestation. The NICU team will be on standby during delivery. Postnatally, the neonate is transferred to the Pediatric Surgery department, with surgery anticipated within three days after birth or as an emergency procedure if indicated. A male infant was delivered by cesarean section at 37 weeks of gestation, with a birth weight of 3140 g, length of 47 cm, and Apgar scores of 9, 10, and 10 at 1, 5, and 10 min, respectively. No abnormal findings were noted in the newborn’s external appearance. Postnatal abdominal ultrasound in the newborn revealed a large, irregular cystic mass measuring approximately 115 × 52 mm within the abdominal cavity. Multiple septa resembling intestinal walls were observed inside the mass, giving it a honeycomb-like appearance. Color Doppler flow imaging (CDFI) revealed only sparse blood flow signals within the cyst wall. The intestinal tract was displaced posteriorly, without notable dilation, and peristalsis remained relatively normal. No significant anechoic fluid collections were observed in the abdominal cavity. The ultrasound findings indicated a giant cystic mass in the abdominal cavity of uncertain origin, with an intestinal duplication cyst considered a possible diagnosis. Additional diagnostic workup was recommended. Postnatal multi-slice spiral CT of the newborn revealed a giant cystic lesion with calcification in the right mid-lower abdomen. Differential diagnoses included meconium pseudocyst secondary to meconium peritonitis and intestinal duplication cyst.

**Figure 5:**
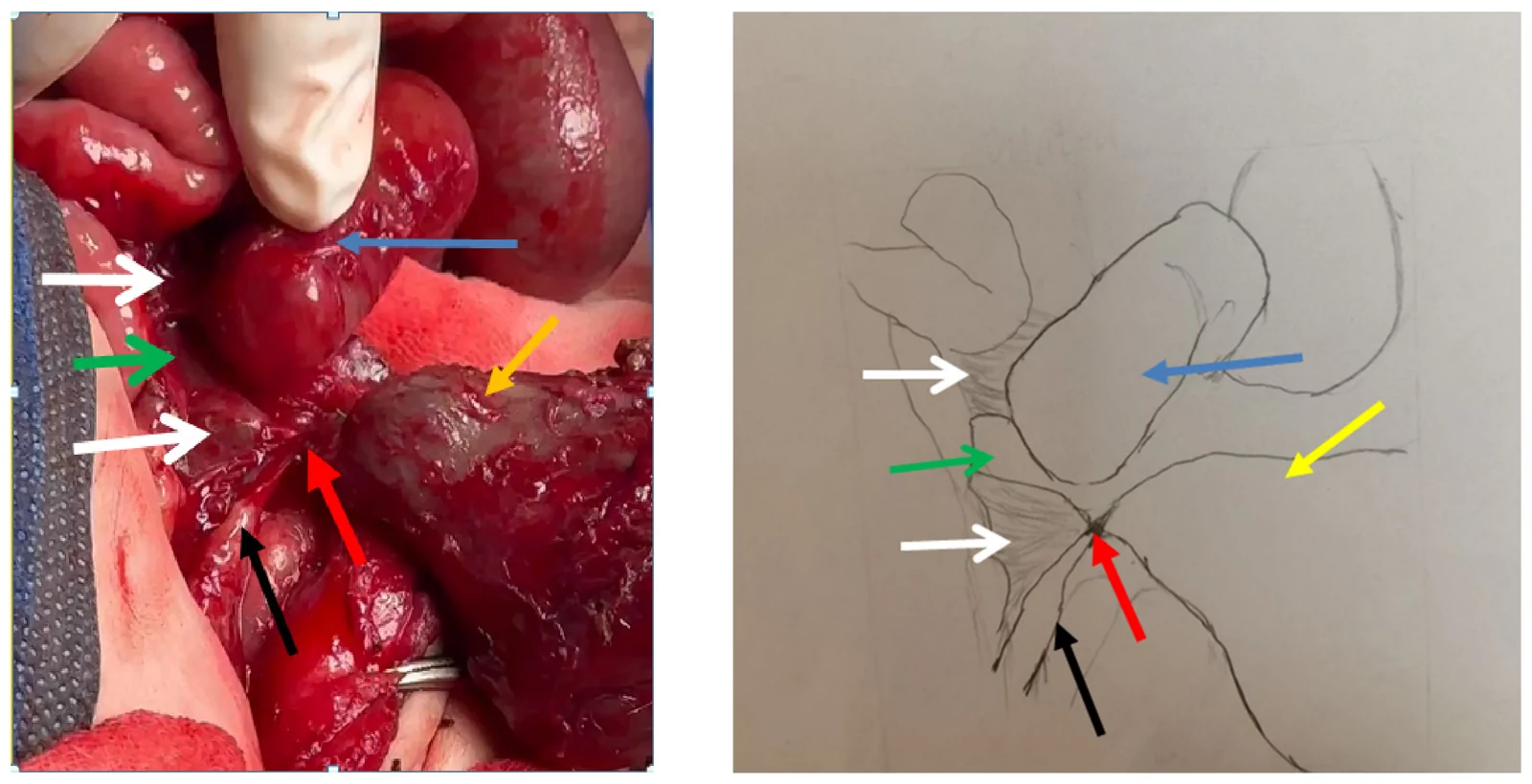
Surgical findings: a cystic abdominal mass (yellow arrow) was located at the terminal ileum and was connected to the distal blind end of the atretic intestine (red arrow). Atresia was present in both the proximal and distal intestine, with the blind ends separated. The mesentery (white arrow) was interrupted, and a mesenteric defect was visible (green arrow). The proximal intestine was dilated (blue arrow), while the distal intestine was narrowed (black arrow).

**Figure 6:**
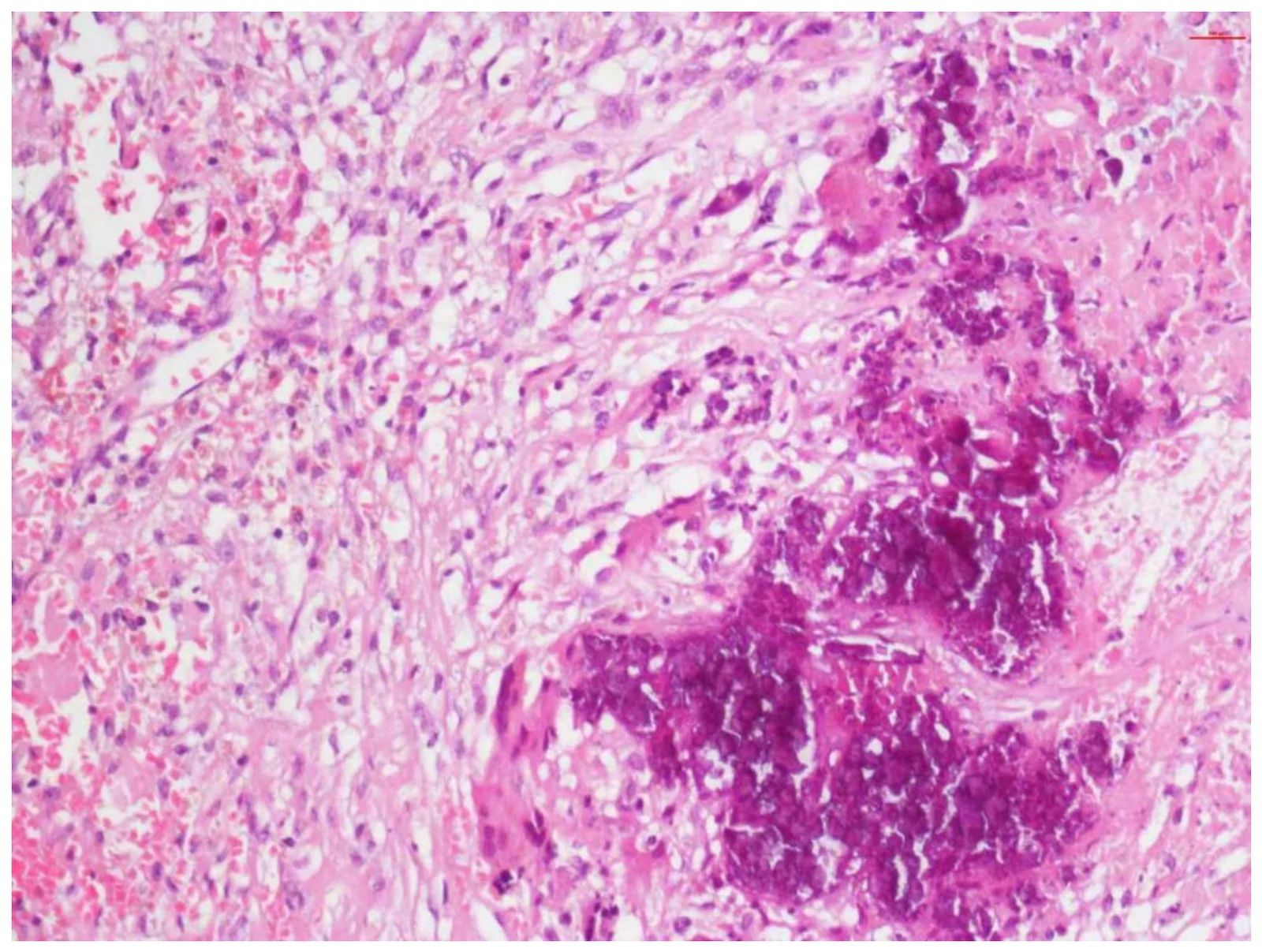
Pathological findings: Hematoxylin-eosin staining at original magnification ×200, demonstrating Necrosis, fibrosis, calcification of the intestinal wall, and multinucleated giant cell reaction.

On the second day after birth, surgical treatment was performed in our hospital. Intraoperative findings revealed a cystic abdominal mass with significant adhesions to the abdominal wall, the inferior margin of the liver, and the intestinal loops. The cystic mass, located at the terminal ileum, resulted from fibrotic encapsulation of a torsed and necrotic intestinal segment. Upon incision of the cystic wall, a large amount of meconium gushed out. Both the proximal and distal intestinal segments were atretic and formed clearly separated blind ends. The mesentery was interrupted. The proximal segment was dilated, with preserved blood supply and sufficient length, while the distal segment was atretic and narrow. The cystic mass, connected to the distal blind end of the atretic intestine, was located only 5 cm from the ileocecal junction. End-to-end anastomosis of the proximal and distal intestinal segments was performed, and the mesenteric defect was repaired. Corresponding intraoperative images are presented (Figure 5). Laparoscopic adhesiolysis, partial enterectomy, and enteroanastomosis were performed. The postoperative diagnosis was small intestinal atresia (type IIIa) complicated by volvulus. The abdominal mass resected during surgery was sent for pathological examination. Microscopic findings revealed complete necrosis of the intestinal mucosa, with fibrosis, calcification, and detachment; only residual shadows were visible locally. The partial intestinal wall shows multinucleated giant cell reaction, vascular proliferation and congestion, accompanied by chronic inflammatory cell infiltration. No evidence of malignancy was found (Figure 6). Pathological Diagnosis: “Abdominal Mass”—consistent with intestinal necrosis accompanied by inflammatory reaction changes.

The patient recovered well postoperatively and was discharged two weeks after surgery. On the second day after birth, a neonatal echocardiogram revealed a patent ductus arteriosus, left-to-right shunting at the atrial level via a patent foramen ovale, and normal global left ventricular systolic function. Follow-up echocardiograms at 1.5 and 4.5 months of age revealed a patent foramen ovale and normal global left ventricular systolic function. The child’s postoperative changes in height and weight are shown in Table 1. The child is currently growing and developing well.

## 3. Discussion

Small bowel atresia is a congenital obstruction of the lumen of the duodenum, jejunum or ileum. Small bowel atresia is one of the most common causes of congenital bowel obstruction, with an incidence between 0.57 and 6.6 per 10,000 live births [1,2]. The most accredited hypothesis relates to an early mesenteric vascular accident leading to ischemia and obliteration of the intestinal lumen [3]. Intestinal volvulus arises when loops of the intestine become twisted around the pedicle of the superior mesenteric artery [4]. The majority of intrauterine volvulus cases occur without any predisposing factors. Less commonly, intrauterine volvulus can be attributed to conditions such as malrotation, intestinal atresia, meconium ileus (associated with cystic fibrosis), or anomalies in the mesentery [5].

Pathological classification depends on the location and morphology of the small intestinal atresia. The Grosfeld modified method [6] is currently the most commonly used system. Type I: Septal atresia, where the intestinal lumen is obstructed by a septum, while the intestinal tube and mesentery remain continuous; the septum may contain pinhole-sized pores, though in rare cases these pores are located at its edge. Type II: Blind end atresia. The intestinal segments at both ends of the atresia form blind pouches, with a fibrous cord connecting them. The mesentery remains intact. Type IIIa: Blind end atresia with mesenteric separation, characterized by blind pouches at both ends and a V-shaped mesenteric defect between them. Type IIIb (ApplePeel atresia): the atresia is located at the proximal jejunum, with the two blind ends separated. The superior mesenteric artery is abnormally developed; only the first jejunal branch and the right colonic artery persist, or the ileocolonic artery serves as the sole nutrient vessel for the distal small intestine beyond the atresia. Type IV involves multiple atresias of the small intestine, which may coexist with types I, II, and IIIa, and the location and number of atresias vary. In this case, atresia occurred at both the proximal and distal ends of the small intestine. The atretic ends showed a blind-pouch configuration, and a mesenteric defect was noted between the two blind ends, resulting in a postoperative diagnosis of type IIIa atresia. Whether the type of intestinal atresia is associated with the occurrence of intestinal volvulus remains unclear, as no specific type has been reported to be more prone to this complication. This issue warrants validation through large-sample data. Due to the retrospective nature of this case, the prenatal ultrasound examination did not demonstrate a clear “whirlpool sign.” The proximal intestine was dilated, whereas the distal segment appeared collapsed upon retrospective image analysis. Therefore, it is hypothesized that intestinal atresia occurred first. The resulting obstruction caused the proximal intestine to become dilated. As the disease progressed, worsening dilation compromised mesenteric stability and potentially triggered intestinal volvulus. This secondary event produced a cystic abdominal mass resembling the “coffee bean sign” on retrospective image analysis, a hallmark of intestinal volvulus. Volvulus can induce ischemic necrosis of the intestine, leading to secondary intestinal atresia, which ultimately resulted in atresia at both ends of the small intestine. This sequence may explain the development of type IIIa small intestinal atresia in this case. Nevertheless, the mechanisms underlying small intestinal atresia and volvulus are highly complex, particularly within the intrauterine environment, making it difficult to ascertain the precise etiology. These two conditions may act as both cause and effect of one another, or they may simply coexist.

In this case, no abnormalities were detected during the mid-trimester ultrasound examination. At 31 weeks of gestation, ultrasonography revealed gastric enlargement, dilated small bowel, and colonic dilatation (later confirmed postoperatively as ileal dilatation). The relatively late gestational age at which these abnormalities were identified may be related to the developmental progression of the digestive tract and the site of obstruction. In this instance, the intestinal atresia with malrotation was located at the ileocecal region, specifically involving the distal ileum. The author initially misinterpreted the findings as colonic dilatation during the 31-week ultrasound, possibly because the obstruction near the ileocecal valve caused the dilated bowel loops to appear “C-shaped” and positioned along the abdominal wall, resembling typical features of colonic dilatation. The honeycomb-like mass observed in the lower abdomen at 31 weeks was, upon retrospective analysis, considered to represent the collapsed colon. When proximal intestinal obstruction occurs, the content of the distal normal bowel decreases, leading to luminal collapse. At 34 weeks of gestation, fetal abdominal MRI revealed a large cystic mass in the mid-to-lower abdomen. This provided useful information regarding the lesion’s size and nature. However, intestinal atresia and volvulus were not considered, likely due to the examiner’s limited experience with these conditions. Due to the relatively low acceptance of fetal MRI among pregnant women and its higher cost compared to ultrasound, this modality is currently less commonly used in our hospital for examining other fetal organ systems, except for more frequent applications in the neurological system, such as cranial imaging. Consequently, our accumulated experience with these diseases remains quite limited. In contrast, ultrasound is a real-time dynamic imaging modality widely accepted by pregnant women during the fetal period. At 35 weeks of gestation, repeat ultrasonography showed no obvious stomach distension, while the small bowel remained dilated. The elongated, dilated bowel loop previously observed in the lower abdomen was no longer clearly visible, having been replaced by a large cystic mass with dimensions consistent with those detected on MRI. Based on the ultrasonographic findings at 31 weeks of gestation, the cystic mass was initially interpreted as a dilated bowel loop. However, intestinal volvulus was not considered, likely because the examiner was insufficiently familiar with its characteristic “coffee bean” sign. Further analysis revealed that the mass comprised tightly apposed dilated bowel loops; the edematous, thickened inner walls of adjacent segments formed a “coffee bean”-like structure with pronounced inner and thin outer walls. The stomach initially enlarged at 31 weeks and then decreased in size by 35 weeks. It is hypothesized that rapid dilatation of the intestinal loops in the abdominal cavity caused the initial gastric enlargement, followed by bowel volvulus that led to the formation of a large cystic mass. This mass subsequently relieved pressure on the stomach, allowing it to decrease in size. Based on a retrospective analysis, the authors hypothesized that fetal intestinal volvulus had already occurred by the time of the MRI examination at 34 weeks of gestation. Accordingly, the ultrasonographic findings at 35 weeks also changed, revealing a so-called cystic mass. Postnatal surgical pathology later confirmed that this cystic mass corresponded to a dilated intestinal segment that had undergone torsion and necrosis. Wax et al. found that the ultrasound manifestations of jejunum atresia follow a certain sequence. The earliest manifestation is the discovery of strong intestinal echoes in the second trimester, followed by typical intestinal or gastric dilation in the second and third trimesters, and finally polyhydramnios in the third trimester. Based on this result, they believe that 28–32 weeks of gestation is the best time to diagnose cases suspected of small intestinal atresia [7]. The results of the present case are similar to those of Wax et al. Fetal intestinal volvulus is a dynamic and evolving pathological process, with sonographic features that vary according to the stage of the condition. Common nonspecific ultrasonographic findings include intestinal dilatation, the “coffee-bean sign” [8], ascites, intraperitoneal calcifications, diminished or absent bowel motility, unclear bowel wall architecture, and polyhydramnios. The “coffee bean sign” is another indicator for diagnosing intestinal torsion and is typically observed in closed-loop intestinal torsion, where the distended loop assumes an elliptical shape and the thickened intestinal walls converge along a central dense line [9]. In the present case, the examiner lacked sufficient awareness of this sign, which precluded a definitive prenatal diagnosis of intestinal volvulus.

Fetal intestinal atresia with volvulus case is extremely rare. Prenatal diagnosis of intestinal atresia is frequently characterized by bowel distension and can be accompanied by meconium peritonitis [10]. When an abdominal cyst is identified, differentiation from a meconium pseudocyst is necessary. A meconium pseudocyst develops when fetal intestinal perforation causes meconium to leak into the peritoneal cavity, where it becomes encapsulated by intestinal loops or the greater omentum. Its typical imaging feature is punctate or linear hyperechoic spots along the cyst wall, resulting from dystrophic calcification. In this case, postpartum CT revealed a large cystic mass with calcification in the right lower abdomen. Given the presence of a cystic mass with calcification, which closely resembles a meconium pseudocyst secondary to meconium peritonitis, the CT examiner considered the differential diagnosis between a meconium pseudocyst caused by meconium peritonitis and an enteric duplication cyst. However, Ultrasound revealed no obvious peritoneal effusion or intra-abdominal calcifications, suggesting that intestinal perforation had not occurred, thereby excluding meconium peritonitis and meconium pseudocyst. During surgery, however, extensive intra-abdominal adhesions were found, attributed to necrosis and inflammatory exudation of the intestinal wall following small bowel volvulus. This was consistent with the pathological diagnosis of an “abdominal mass,” which confirmed intestinal necrosis accompanied by inflammatory changes. When an abdominal cyst is identified, differentiation from small intestinal duplication (cystic type) is also necessary. A cystic duplication cyst of the small intestine usually appears as a round or oval cyst adjacent to the intestinal loop, often lacking communication with the intestinal lumen and typically located within the mesentery. The cyst wall resembles that of a normal gastrointestinal tract. Compression of adjacent bowel loops may cause intestinal dilation or obstructive symptoms, and a definitive diagnosis relies on postoperative pathology. In this case, the cystic mass detected at 34 weeks’ gestation showed features consistent with intestinal wall structure and bowel dilation, prompting the MRI examiner to initially suspect intestinal duplication. Small intestinal atresia must also be distinguished from congenital megacolon (total colonic type). Total colonic aganglionosis manifests with signs of low small bowel obstruction, such as generalized fluid accumulation and dilation of the small intestine, alongside a notably narrowed and collapsed colon. These imaging features closely mimic those of small intestinal atresia, requiring further postnatal assessment to establish a definitive diagnosis. Therefore, the ultrasound examiner also raised the possibility of congenital megacolon in this case during the prenatal period. The imaging diagnosis in this case was markedly challenging both before and after birth, highlighting the importance of a comprehensive understanding of this condition.

The incidence of chromosomal abnormalities in fetuses with small intestinal atresia or stenosis is significantly lower than in those with other gastrointestinal malformations. Therefore, routine karyotype analysis is not recommended for isolated cases of small intestinal stenosis or atresia. However, in approximately 30% of the cases, a chromosomal abnormality is noted, mainly trisomy 21 [11]. The risk of chromosomal abnormalities is elevated in fetuses that also present with abnormalities in other organ systems. In the present case, the pregnant woman underwent amniocentesis for genetic testing, and the results of chromosomal microarray (CMA) combined with methylation analysis and CMV-DNA were normal. At 35 weeks of gestation, ultrasound revealed an increased cardiothoracic ratio, mild tricuspid regurgitation, elevation of the diaphragm, and bilateral hydrocele of the tunica vaginalis. These findings were attributed to the mass effect within the abdominal cavity and the resulting increase in intra-abdominal pressure. After birth, these secondary changes resolved, and serial postnatal follow-up confirmed their transient.

The primary treatment for this condition is surgical intervention. However, no consensus has been reached on the optimal timing of delivery for affected fetuses. The prognosis of fetal intestinal volvulus depends on multiple factors, including gestational age, birth weight, the extent of necrotic bowel, and associated abnormalities [5]. Therefore, accurate diagnosis of fetal intestinal volvulus, selection of appropriate delivery timing, and timely surgical intervention are all crucial [12]. Continuous ultrasound monitoring is necessary to assess bowel condition and determine whether termination of pregnancy is warranted [13]. However, the complications associated with in utero intestinal atresia are poorly documented, and the need for prompt intervention is not widely acknowledged [14]. Some viewpoints suggest that fetuses with a gestational age exceeding 34 weeks should undergo immediate delivery upon ultrasound evidence of bowel deterioration. For fetuses younger than 34 weeks with normal fetal movement and fetal heart monitoring, pregnancy can be continued with intensified monitoring [9,15]. Galani A et al. [11] reported a case of fetal jejunal atresia in which an ultrasonography at 33 weeks of gestation revealed fetal intestinal dilatation. Due to a history of previous cesarean section, an elective cesarean delivery was performed at 34 weeks of gestation. The neonate subsequently underwent surgical intervention, and the prognosis was favorable. Similarly, in a case of fetal intestinal atresia reported by Zeng X [13], a pregnant woman presented with decreased fetal movements at 32 weeks of gestation. Fetal ultrasound demonstrated a “whirlpool sign,” and both ultrasound and magnetic resonance imaging (MRI) suggested small bowel volvulus and obstruction. An emergency cesarean section was performed, followed by urgent neonatal surgery, resulting in a good prognosis. Furthermore, in a case of fetal ileal atresia with dilatation and volvulus reported by Toyama C [14], the pregnant woman presented with decreased fetal movements at 35 weeks of gestation. Fetal ultrasound showed intestinal dilatation and a whirlpool sign. An emergency cesarean section was performed, and the neonate received surgical treatment, with a favorable outcome. In this case, ultrasound at 31 weeks of gestation revealed gastric dilation and intestinal dilatation, while at 35 weeks, a cystic abdominal mass was detected alongside polyhydramnios. Throughout this period, fetal movement and heart rate monitoring remained normal, and the fetal condition was stable. Cesarean section was therefore performed at 37 weeks of gestation. On the second day after birth, the neonate underwent surgery and achieved a favorable prognosis. In these cases, favorable outcomes were achieved by selecting the appropriate delivery timing and surgical approach. In all cases, fetal abnormalities were detected via ultrasound during the late trimester, specifically after 30 weeks of gestation. Nevertheless, the novelty of the present case lies in the detection of fetal intestinal atresia at 31 weeks of gestation using ultrasound. Under continuous ultrasound monitoring, with normal fetal movement and fetal heart monitoring, the pregnancy was deliberately prolonged to full term (37 weeks). This approach carries important clinical significance by reducing the incidence of preterm birth, minimizing prematurity-related complications, and ensuring surgical tolerability and postoperative recovery in affected neonates. Therefore, the authors recommend that delivery timing in such cases be determined through a balanced risk–benefit assessment with individualized decision-making.

## 4. Conclusions

Based on the retrospective analysis of this case, we propose that serial prenatal ultrasound and recognition of progressive imaging changes—from intestinal atresia to volvulus—carry important clinical significance. Multidisciplinary prenatal consultation helps formulate feasible postpartum treatment plans, thereby improving clinicians’ ability to manage such conditions effectively.

## Figures and Tables

**Table 1:** Postoperative Changes in Height and Weight of the child.

Index	Age (Month)					
	0.87	1.53	3.73	4.97	13	18
Weight (kg)	3.2	3.75	6.1	6.8	9.3	10.05
Height (cm)	50	52.4	62	67	77	82

## Data Availability

The authors declare that the data for this research are available from the corresponding authors upon reasonable request.

## Abbreviations

The following abbreviations are used in this manuscript:

MRI	Magnetic resonance imaging
CT	Computerized tomography
STO	stomach
Bo	bowel
LK	left kidney
BL	bladder
M	mass
SP	spleen
NIPT	Non-invasive prenatal testing
NT	Nuchal translucency
CDFI	Color Doppler Flow Imaging
AFI	Amniotic fluid index
CMA	Chromosomal microarray analysis
CMV-DNA	Cytomegalovirus deoxyribonucleic acid
Trio–WES	Trio–Whole exome sequencing
MDT	Multidisciplinary team
NICU	Neonatal Intensive Care Units
